# Reactive Arthritis in a 37-Year-Old Female With SARS-CoV2 Infection

**DOI:** 10.7759/cureus.9698

**Published:** 2020-08-12

**Authors:** Zach Danssaert, George Raum, Somkiat Hemtasilpa

**Affiliations:** 1 Physical Medicine and Rehabilitation, Reading Health System, Reading Hospital, Reading, USA; 2 Physical Medicine and Rehabilitation, Philadelphia College of Osteopathic Medicine, Philadelphia, USA; 3 Physical Medicine and Rehabilitation, Reading Hospital Rehabilitation at Wyomissing, Reading, USA

**Keywords:** keywords: sars-cov-2, reactive arthritis

## Abstract

We report the case of a 37-year-old female who presented for evaluation of acute 10/10 right hand pain, 12 days after testing positive for SARS-CoV2. The patient was admitted to the hospital due to the severity of her pain. As an inpatient, extensive workup by the medicine team and rheumatology revealed no structural, vascular, or neurogenic cause of her pain. The patient's blood work was unremarkable for elevations in lyme serology, antinuclear antibody (ANA), rheumatoid factor, and uric acid. It was determined that the cause of her pain was most likely reactive arthritis (ReA) secondary to her SARS-CoV2 infection. She was treated with voltaren gel, neurontin, and oral dilaudid as needed and discharged. Upon follow-up, her pain improved and she was prescribed a wrist splint, ultram, and occupational therapy for perceived wrist tendinitis. To our knowledge, this is the first description of a case of ReA caused by the SARS-CoV2 virus.

## Introduction

Reactive arthritis (ReA) is an inflammatory arthritis that causes an asymmetric, oligoarticular arthritis one to four weeks after infection with a causative pathogen [1]. The disease commonly impacts genetically susceptible individuals with a strong link to the HLA-B27 antigen, between the ages of 20 and 40 years old [1-2]. Up to 70% of patients with ReA have been determined to have the HLA-B27 antigen [3]. The disease has been found to impact men and women at equal rates, however, men seem to have a higher rate of urethritis [1-2].

The most common triggering pathogens of ReA include *Chlamydia trachomatis*, *Salmonella*, *Shigella*, *Yersinia*, and *Campylobacter*. Other potential organisms include *Escherichia coli*, *Clostridioides difficile*, and *Chlamydia pneumoniae*. While other bacteria and viruses have been proposed, they are not included as causative agents of ReA by definition [4-5]. Well-documented entrance points for organisms leading to ReA include the gastrointestinal tract, urogenital tract, and upper respiratory tract [3]. The clinical presentation is varied, but most often includes musculoskeletal symptoms such as peripheral arthritis, enthesitis, and axial back pain [6]. The condition is also classically associated with genitourinary and ocular symptoms [3]. 

Since late 2019, the COVID-19 pandemic has thrown the medical community into a fervor to explain the clinical manifestations of the disease. SARS-COV2 is a RNA betacoronavirus that has been shown to have a similar receptor binding gene structure to the SARS coronavirus [7]. Clinical manifestations ranging from Kawasaki-like illness to large vessel stroke have been attributed to the virus [8-9]. This report presents a case of ReA secondary to SARS-CoV2 infection. It emphasizes yet another implication and possible sequelae of the novel virus. 

## Case presentation

A 37-year-old female with a past medical history of congestive heart failure, asthma, gastroesophageal reflux disease (GERD), and morbid obesity with a history of bariatric surgery presented with an acute onset of pain and swelling in the right hand. The pain came on suddenly in the morning and progressively worsened throughout the day causing her to drive herself to the ER. The pain and swelling were localized to the dorsal aspect of the right hand extending to the forearm without erythema. She described the pain as a 10/10 intensity, which worsened with palpation and movement. Twelve days prior, the patient tested positive for active SARS-CoV2 after experiencing symptoms of cough, congestion, fevers, chills, and myalgias. She was not hospitalized for SARS-Cov2 infection and her symptoms subsided prior to her right hand pain. She denied symptoms of fever, chills, cough, sputum production, shortness of breath, or chest pain upon presentation. She also denied any trauma to the hand, known tick/insect bites, history of gout, lyme disease, gonorrhea, chlamydia, diarrhea, or history of joint problems. She had not experienced pain like this in the past. Family history was noncontributory for any rheumatological conditions. She denied using any drugs, smoking, or alcohol use. 

**Figure 1:**
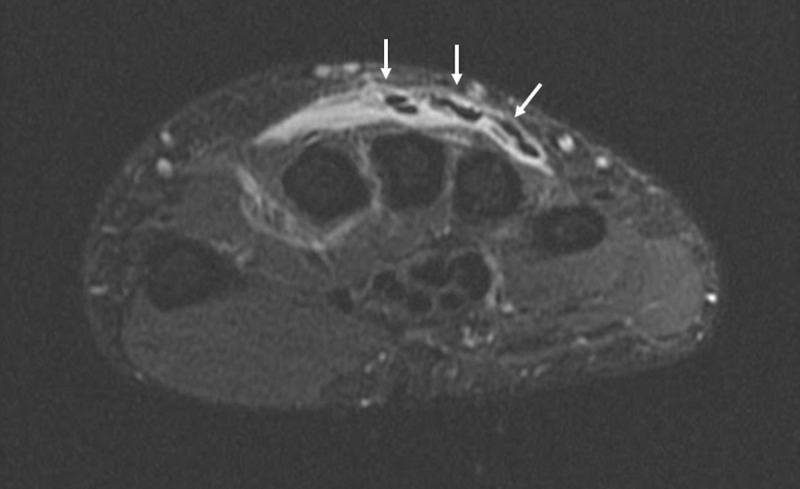
MRI of the right hand with contrast, axial view. Inflammatory edematous changes surrounding the extensor tendons of the second, third, and fourth compartments (white arrows), and to a lesser extent the fifth and sixth extensor compartments. The second, third, and fourth extensor compartments with mild synovial enhancement involving the tendon sheaths.

While in the ER, she was afebrile and hemodynamically stable. Her right hand and phalangeal joints had full range of motion, but were tender to palpation, with mild swelling, on the dorsal hand extending to the forearm. Laboratory workup was within normal limits for C-reactive protein (CRP), lactic acid, and erythrocyte sedimentation rate (ESR). There was a mild leukopenia and anemia with white blood cell count of 3.5 and hemoglobin of 11.4. Blood culture of two separate samples was negative. An MRI of the right upper extremity showed inflammation around the extensor tendons of the second, third, and fourth compartments with associated mild synovial enhancement of the tendon sheaths suggestive of tendonitis (Figure 1). No drainable abscess was identified. Doppler ultrasound of the right upper extremity was negative for deep vein thrombosis (DVT). The patient was given dilaudid IM, oxycodone p.o., and lidocaine patch with minimal improvement in pain and tenderness. She was then admitted by the hospitalist for further workup. Lyme serology, antinuclear antibody (ANA), rheumatoid factor, uric acid level, and repeat COVID-19 test were completed. All labs were negative except for ANA, which was speckled. She refused chlamydia/gonorrhea testing because she was on her menstrual period, and she stated that she was regularly screened for sexually transmitted diseases (STDs) with consistent negative test results. The patient denied recent vaginal discharge or dysuria at the time of admission. A rheumatologist was consulted who assessed the patient and commented that rheumatoid arthritis or crystalline disease was less likely, and a monoarticular arthritis as a reaction to COVID19 was the likely cause of her symptoms. The patient did not receive nonsteroidal antiinflammatory drugs (NSAIDs) or steroids because of recommendation from her bariatric surgeon to decrease the risk of gastric ulcers. The patient was discharged from the hospital with voltaren gel, neurontin, and oral dilaudid as needed. 

She followed up with a physiatrist two weeks after discharge. Over the two weeks, her pain had improved from a 10/10 intensity in the hospital to a 2/10. However, the patient still complained of intermittent tenderness at the dorsum of the right hand and wrist. She denied weakness, numbness, or tingling of the hand and fingers. She denied symptoms, such as vaginal discharge and dysuria, leading to the belief that she was not currently suffering from a chalmydia or gonorrhea infection. Range of motion of the right hand and digits was somewhat limited due to pain, but without swelling. A right wrist support was prescribed to minimize motion contributing to the tendinitis. The patient followed up with physiatry again two weeks later. Her pain and tenderness to the hand and wrist had improved, however, she still complained of tenderness to the dorsal aspect of the wrist and hand, especially to the finger joints. Musculoskeletal ultrasound of the right hand and wrist demonstrated inflammation surrounding the flexor and extensor tendons of the right hand. The patient was prescribed ultram for pain control, occupational therapy, and asked to follow up if the pain did not improve.

## Discussion

The patient presented with severe wrist pain two weeks after testing positive for COVID-19 disease with associated symptoms. A full workup was done, which excluded other potential causes including traumatic arthritis, septic arthritis, rheumatoid arthritis, crystal-induced arthritis, lyme arthritis, systemic lupus erythematosus, and psoriatic arthritis. Unfortunately, the patient denied chlamydia/gonorrhea testing, but she had a history of negative chlamydia/gonorrhea tests, and she denied vaginal discharge or dysuria at the time of initial presentation and on subsequent follow up. Due to the patient's refusal of STD testing, chlamydia and gonorrhea cannot be completely ruled out. Overall, it was the belief of the consultants regarding this patient's case that the most likely cause of symptoms was sequelae of the SARS-CoV2 virus. While we are still learning about the mechanism and potential complications of the SARS-CoV2 virus, it appears that the virus caused ReA in this patient. 

The clinical criteria for ReA include the patient experiencing arthritis days to weeks following the initial infection, and the arthritis is typically mono- or oligoarticular, generally involving the lower extremities or fingers and tendon insertions [4]. The recommended treatment for arthritis is with NSAIDs, with naproxen or diclofenac, to provide symptom relief [10-11]. Intra-articular steroid injections should be considered in patients who do not respond to NSAIDs [11]. If the patient has prolonged symptoms, disease-modifying antirheumatic drugs (DMARDs) or tumor necrosis factor antagonists (TNF-α) can be considered [11]. If there is an identified bacterial cause, antibiotics have been shown to have a positive impact in some studies [11]. 

The pathogenesis of ReA has many proposed mechanisms, but the most accepted is the arthritogenic hypothesis [3]. This proposes that the microbes persist in the epithelium, lymphoid tissues, liver, and spleen. As the microbes and antigens are disseminated into the joints a localized inflammatory response is triggered. The acute inflammation is followed by a CD4+ T-cell response driving the arthritic process. While unclear, it is also postulated that HLA-B27 cross-reactivity with these antigens makes this process more likely to occur [6]. Indeed, there appears to be an association between HLA-B27 and ReA in Caucasian HIV-infected patients [12].

While the classic picture of ReA has been observed after a bacterial infection, viral causes have been linked to ReA in the past. The HIV has been observed to cause ReA in some patients. The pattern of the disease was first observed in the early stages of the HIV epidemic. Interestingly, in regions where HIV was more likely to be contracted through genitourinary or gastrointestinal means through sexual contact, as opposed to IV means such as IV drug use, ReA was more likely to be observed, much like the bacterial causes [13]. It is unclear whether HIV causes ReA directly or through exposure to other pathogens that infect the patient [14-15]. 

Even though the patient appeared to present with a delayed inflammatory arthritis, we cannot exclude the possibility of a viral arthritis. Viral arthritis generally presents with an acute-onset polyarticular arthritis and has been well documented with parvovirus, alphaviruses, hepatitis B, hepatitis C, Epstein-Barr virus (EBV), Zika virus, and chikungunya virus [16]. Furthermore, acute viral arthritis rarely presents with monoarthritis and often occurs in conjunction with other viral symptoms like fever, rash, and lymphadenopathy [16]. The delayed response after initial recovery, and localization of the arthritis to just the right hand, decreases the likelihood of a viral arthritis. It is also possible that the patient’s obesity could have contributed to her arthritis.

The presentation and sequelae of the SARS-Cov2 virus has been variable. While this is an example of a delayed reaction, there have been cases where the initial presentation of the virus has been joint pain. One case in particular, the virus was misdiagnosed as dengue fever because of the initial presentation of arthralgia [17]. Similar to the proposed mechanism of ReA, multisystem inflammatory syndrome in children (MIS-C) is thought to be a postinfectious process following SARS-Cov2 infection [8, 18]. It appears that an abnormal immune response leads to MIS-C, which further supports the possibility that there can be inflammatory reactions after the virus has been cleared.

While the body of literature investigating the SARS-CoV2 virus is rapidly expanding, after an extensive literature search, this is the first written report of the presentation of ReA post SARS-CoV2 infection to our knowledge. This case represents an additional clinical scenario to be cognizant of when treating patients after they recover from a SARs-CoV2 infection. 

## Conclusions

There is a lack of documentation of the SARS-CoV2 virus leading to ReA. We have presented the case of a patient who developed severe right wrist pain and swelling after a positive COVID-19 test two weeks prior. It is possible that the patient’s symptoms could have been unrelated to COVID-19, but the extensive workup in the hospital did not reveal another potential cause. Further documentation is needed to explore the relationship between the SARS-CoV2 virus and ReA.
